# Genomic Survey of Selection Footprints in Three Buffalo Breeds from Eastern Europe

**DOI:** 10.3390/ani16101529

**Published:** 2026-05-16

**Authors:** Medhat S. Saleh, Abdelfatah R. Zaghloul, Mayra Gómez Carpio, Claudia Pierini, Pasquale De Palo, Vincenzo Landi

**Affiliations:** 1 Department of Veterinary Medicine, University of Bari Aldo Moro, 70010 Valenzano, BA, Italy; medhat.elshahat@uniba.it (M.S.S.); c.pierini@phd.uniba.it (C.P.); pasquale.depalo@uniba.it (P.D.P.); 2 Department of Animal Production, Faculty of Agriculture, Benha University, Benha 13736, Egypt; abdelfatah.rashed@fagr.bu.edu.eg; 3 Italian National Association of Buffalo Breeders, 81100 Caserta, Italy; m.gomezcarpio@anasb.it

**Keywords:** buffalo, signatures of selection, genomic regions, candidate genes, SNPs

## Abstract

The buffalo is an important agricultural species due to its many productive characteristics, which encourage its use worldwide. Uncovering the processes of selective sweeps is critical for a comprehensive understanding of genomic mechanisms that influence phenotypic differentiation in buffalo. This study aims to refine signatures of selection in Bulgarian (BUL), Hungarian (HUN), and Romanian (ROM) buffalo breeds using runs of homozygosity (ROHs), the integrated haplotype score (iHS), the standardized log-ratio of the integrated site-specific extended haplotype homozygosity (EHH) between pairs of breeds test (Rsb), and cross-population EHH (XP-EHH) approaches. The SNP dataset of 160 genotypes from BUL, HUN, and ROM buffalo breeds was genotyped using the Axiom^®^ Buffalo Genotyping Array 90K from Affymetrix. By combining the ROH, iHS, Rsb, and XP-EHH methods, we identified many important genomic regions and candidate genes associated with milk production (*SLC24A2*, *TMEM132C*, and *ALCAM*), reproduction (*CSMD1*, *NTS*, *PLIN2*, *GPC5*, and *FSHR*), growth (*MYOM2*, *CLN8*, and *RRAGA*), immune response (*METTL25*, *MLLT3*, *NAALADL2*, and *GAB2*), and adaptation (*ADAMTSL1*) in BUL, HUN, and ROM buffalo breeds. Our findings highlighted selection signals and genes related to important economic traits in the BUL, HUN, and ROM buffalo breeds, providing promising candidate genes for further research and inclusion in conservation and selection plans for these breeds.

## Simple Summary

Understanding the genetic basis of economically important traits in buffalo is essential for conservation and selection strategies. A total of 160 genotypes from buffalo breeds in Bulgaria, Hungary, and Romania were used to identify signatures of selection. The results revealed several potential candidate genes within genomic regions under positive selection. These genes are associated with production traits. These insights can be applied to conservation and breeding programs to support the sustainable production of buffalo breeds.

## 1. Introduction

The main historical migration route for river buffalo from Asia into Europe has been through Eastern European countries. Riverine buffalo were domesticated in India and spread to Southwestern Asia, Egypt, and Turkey before arriving in Eastern Europe and Italy in the seventh century. It is likely that some of these animals returned to Egypt, Turkey, and Bulgaria with the Crusaders and expanded throughout the Balkans throughout the 12th century [1]. The genome of water buffalo consists of approximately 2.66 Gb [2]. There are two types of domesticated water buffalo, the river buffalo (*Bubalus bubalis*, 2n = 50) and the swamp buffalo (*Bubalus carabanesis*, 2n = 48) [3].

The Bulgarian Murrah is an officially recognized breed in Europe for draft, meat, and dairy products. The native *Bulgarian buffalo* breed has held considerable importance in Europe. Since 1962, buffalo of the indigenous Bulgarian breed have been extensively crossbred with Murrah bulls and then especially selected to produce dairy animals with a high milk fat content [4]. The average milk production during the standard lactation period for the Bulgarian Morah breed in Bulgaria was 2245.37 kg, with an average milk fat content of 7.77% and milk protein content of 4.34% [5]. The body weight of an adult male is 700 kg and that for an adult female is 600 kg [4]. According to the annual agricultural reports of the Bulgarian Ministry of Agriculture and Food (2016–2024), the number of buffalo has been increasing annually since 2016, reaching 14,000 heads, and the quantity of buffalo milk produced has increased accordingly [6]. The Romanian buffalo breed is related to the common domestic buffalo (*Bubalus bubalis*) and the wild Indian buffalo (*Bubalus arnee*). Genetically and ecologically, it is a Mediterranean river buffalo [7]. The buffalo was introduced to Romania with the invasion of the Carpathian and Danube regions by the Huns and Avars. The buffalo found suitable agricultural climatic conditions, and thus, in Romania, a herd of buffalo arose that is completely different from other similar herds, which have their own evolutionary path as a result of reproductive isolation [8]. The majority of Romanian buffalo are of the Carpathian variety, which has a great genetic resource and is well adapted to the cold environment [8]. Romanian buffalo have excellent traction, meat, and milk qualities. The Carpathian buffalo is distinguished by its length, with a waist circumference of 132 cm, a relatively short trunk length of 139 cm, a small body size of 107%, a shallow chest with a depth ratio of 54%, a medium-sized udder, longer and thicker limbs at 21 cm, and an appearance closer to that of a draft animal [7]. In contrast, the Danube buffalo is distinguished by its short stature, with a waist circumference of 129 cm, a rectangular body shape of 110%, a deeper chest with a depth ratio of 55%, a more developed udder, thinner limbs, and a drier appearance [7]. Romanian buffalo thrive under optimal breeding, exploitation, and management circumstances. Accordingly, the production of milk (1300–2000 kg/lactation, fat 100–140 kg, and protein 60–75 kg) and average daily weight gain (600–800 g/day) have been reported [3]. According to the FAO report for 2020, there are only 14,000 buffalo in Romania [9]. The Transylvanian Buffalo Breeders Association recently announced that the number of female water buffalo in Romania is 16,721 [10]. Strategies exist to improve the situation of this species and have been recently developed and approved by national authorities [10]. The problem with implementing this improvement plan lies in the small size of the farms, the small number of animals, and their relative isolation. In the 16th century, the Turks introduced Mediterranean buffalo to Hungary [11]. The Hungarian buffalo, also known as the water buffalo and domestic buffalo, has been a part of Hungary’s indigenous fauna for generations. Approximately 2000 heads were raised on small farms within national parks as gene reserves and were used for meat production [1].

Over the decades, natural and artificial selection factors, influenced by cold environments and management systems, have contributed to shaping the basic economic characteristics of Bulgarian, Hungarian, and Romanian buffalo breeds. Population genetics theory suggests that functional genes subject to selection reveal distinctive patterns called “selective signatures” [12]. The discovery of these selection signatures related to milk and meat production is critical for gaining a full understanding of economically significant traits and their uses in buffalo breeding programs.

The development of high-density single nucleotide polymorphism (SNP) and low-cost genotyping techniques has made it possible to study genetic diversity and selection fingerprints in livestock species [13]. Different statistics like the integrated haplotype score (iHS) [14] and runs of homozygosity (ROHs) have been used within population analysis [15]. The standardized log-ratio of the integrated site-specific extended haplotype homozygosity (EHH) between pairs of populations test (Rsb) and cross-population EHH (XP-EHH) approaches have been applied for intra-population analysis [16,17]. Understanding the genetic basis of responses to selection and local adaptation in BUL, HUN, and ROM breeds is important for conservation strategies and selection programs for productive traits. Previous studies have focused primarily on genetic diversity, population structure, and relatedness between the BUL, HUN, and ROM buffalo populations in Eastern Europe and buffalo populations worldwide [18,19], whereas this study focuses on identifying selection signals using multiple haplotype-based and homozygous fragment methods, integrating overlapping regions to find potential candidate genes for economically important traits. Therefore, the current study aimed to reveal the genetic diversity between BUL, HUN, and ROM buffalo populations and to identify selection signatures within their genomes using ROH, iHS, Rsb, and XP-EHH methods.

## 2. Materials and Methods

### 2.1. Animals and Genotyping Data

The genotyping data of 160 water buffalo from three Eastern European countries (Bulgaria, Hungary, and Romania) were used in this study. The genomic information of these genotyping data was obtained from the Dryad repository (https://doi.org/10.5061/dryad.9cnp5hqgc, accessed on 8 April 2021). Three buffalo breeds were used in this study: 58 samples of the Bulgarian Murrah Buffalo population (BUL) were collected from two farms located in Schumen and Veliko Tarnovo areas in Bulgaria; 55 samples of Hungarian Buffalo breeds (HUN) from three farms located in the Csákvár, Földes, and Tiszatáj areas in Hungary; and 47 samples of Romanian Buffalo breed (ROM) were collected from Șercaia farm in Brașov County, Romania [19]. The quality control and genotyping using the Axiom^®^ Buffalo Genotyping Array 90K from Affymetrix were performed by ATLAS Biolabs GmbH (Berlin, Germany). Allele calling was carried out using Axiom Analysis Suite software V4.0.1 (Applied Biosystems by Thermo Fisher Scientific, Waltham, MA, USA) following the pipeline for the Affymetrix Axiom genotyping workflow [20] and using the reference buffalo genome assembly (UOA_WB_1) [2].

### 2.2. Quality Control

PLINK v.1.9 software was used to conduct quality control (QC) and filtering [21]. Only the 24 autosomes were retained, while sex chromosomes and mitochondrial markers were excluded. SNPs with a call rate lower than 95%, SNPs with a minor allele frequency (MAF) lower than 5%, and animals with larger than 10% of missing genotypes were excluded. After filtering, 60,327 SNPs and 160 samples passed filters and QC.

### 2.3. Principal Component Analysis and Admixture

Prior to principal component analysis (PCA) and admixture analysis, Linkage disequilibrium pruning was performed using PLINK 1.9 [21], with a 50 SNP window size, a 5 SNP step size, and an R^2^ threshold of 0.60, resulting in 45,161 SNPs. PCA was performed to detect the genetic relationship between breeds using PLINK v.1.9 software [21]. ADMIXTURE v1.3 software was used to analyze the population genetic structure using K from 2 to 7 [22]. The optimal K value applied according to the cross-validation error (CV) and the BITE v2 R package was used to visualize the graph [23].

### 2.4. Detection of Selection Signatures

The ROH, iHS, Rsb, and XP-EHH approaches were applied to detect selection signatures in the three Eastern European buffalo breeds. The ROH and iHS methods were used to identify recent selection events within breeds, while the Rsb and XP-EHH methods were used to assess pairwise comparisons between BUL, HUN, and ROM breeds.

### 2.5. Run of Homozygosity Analysis

Runs of homozygosity (ROHs) were detected using PLINK v1.9 [21], with the following parameters, as described by Noce et al. [19]: --homozyg-kb 1000, --homozyg-window-missing 2, --homozyg-window-threshold 0.05, --homozyg-window-het 1, --homozyg-window-snp 50, --homozyg-snp 50, --homozyg-density 50, and --homozyg-gap 250. To identify selection signatures in regions of extended homozygosity “referred to as ROH islands”, the frequency of each SNP within ROH segments was calculated as the number of times an SNP appeared within an ROH, divided by the total number of individuals in each group [24]. The top 1% of SNPs with the highest ROH incidence in each breed was used as a threshold to define candidate regions under selection. These ROH islands were visualized using the ‘qqman’ package in R v4.6.0 [25].

### 2.6. The iHS, Rsb, and XP-EHH Analyses

The three extended haplotype homozygosity (EHH)-based statistics—iHS, Rsb, and XP-EHH—were computed using the REHH R package [26]. Before these analyses, genotype phasing and imputation of missing data were performed using Beagle v5.0 with default parameters [27]. The iHS method is based on comparing the decay of extended haplotype homozygosity between reference (allele 0) and alternative (allele 1) alleles at each SNP, as originally described by Voight et al. [28]. Therefore, the trend in iHS values should be interpreted with caution, as positive or negative scores do not necessarily indicate true ancestral or derivative alleles. The standardized iHS value was calculated using the following formula:

$$iHS=\frac{In\left(\frac{{iHH}_{A}}{{iHH}_{D}}\right)-E_{p}\left[In\left(\frac{{iHH}_{A}}{{iHH}_{D}}\right)\right]}{{SD}_{p}\left[In\left(\frac{{iHH}_{A}}{{iHH}_{D}}\right)\right]}$$

where ${iHH}_{A}$ and ${iHH}_{D}$ denote the EHH score for ancestral and derived core alleles, respectively. $E_{p}\left[In\left(\frac{{iHH}_{A}}{{iHH}_{D}}\right)\right]$ and ${SD}_{p}\left[In\left(\frac{{iHH}_{A}}{{iHH}_{D}}\right)\right]$ represent the expectation and standard deviation within the frequency bin *p*.

The iHS scores were transformed into two-sided *p*-values using the formula piHS  =  −log10[1 − 2|Φ(iHS) − 0.5|], where Φ(iHS) is the cumulative Gaussian distribution function of iHS [21]. piHS values can be defined as −log 10 (−*p* value), considering that iHS values are normally distributed under neutrality. The iHS candidate regions were defined as those located within the top 1% iHS scores and containing at least 10 neighboring SNPs exceeding the threshold within Mb sliding windows overlapping by 20 Kb, and the highest |iHS| value within each window was used as the test statistic.

The Rsb and XP-EHH scores were estimated between breeds according to [11], respectively. As in iHS, assuming that Rsb and XP-EHH values are normally distributed, SNP scores were further transformed into two-sided *p*-values: pRsb = −log10[1 − 2|Φ(Rsb) − 0.5|] and pXP-EHH = −log10[1 − 2|Φ(XP-EHH) − 0.5|]. Both the Rsb and XP-EHH methods defined candidate regions as those located in the top 1% Rsb and XP-EHH scores containing at least 10 neighboring SNPs exceeding the threshold −log10 (*p*-value) ≥ 3 within 2 Mb sliding windows, nested by 20 Kb. The positive and negative values for Rsb and XP-EHH indicate the strength of selection, with positive values indicating stronger selection in the first breed and negative values indicating stronger selection in the second breed.

### 2.7. Annotation and Enrichment Analysis

The full list of annotated genes for the buffalo genome assembly (UOA_WB_1) was downloaded from the NCBI online database. The official gene symbol was classified within the identified genomic regions using the intersectBed command in BEDTools software v2.31.1 [29].

Furthermore, the DAVID software was used to identify Gene Ontology (GO) terms of the function candidate genes within genomic regions detected using the four methods in Eastern European buffalo breeds. In this study, we used the David 2021 version for GO analysis (https://davidbioinformatics.nih.gov/workspace.html, accessed on 23 March 2022).

## 3. Results

### 3.1. Genomic Relationship and Breed Separation

**Figure 1:**
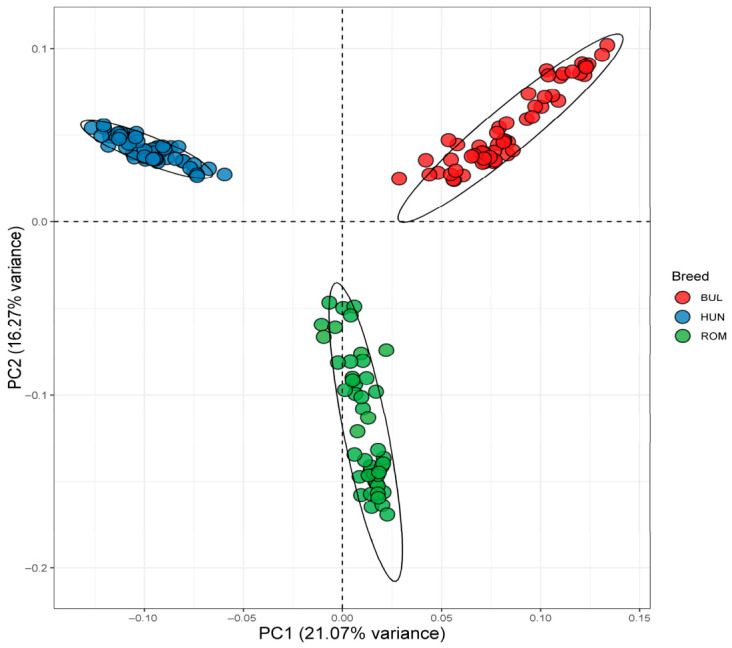
Principal component analysis of three Eastern European buffalo breeds. BUL = Bulgarian; HUN = Hungarian; ROM = Romanian. The ellipse in the PCA plot represents the 95% confidence interval (confidence level = 0.95) for each breed.

The results of PCA between the BUL, HUN, and ROM buffalo populations are presented in Figure 1. The PCA1 explained 21.48% of the overall genetic variance, while the PCA2 accounted for 16.47% of the overall genetic variance. The PCA1 separated the BUL and HUN buffalo breeds from the ROM buffalo breed. The PCA2 separated the BUL buffalo breed from the HUN buffalo breed.

**Figure 2:**
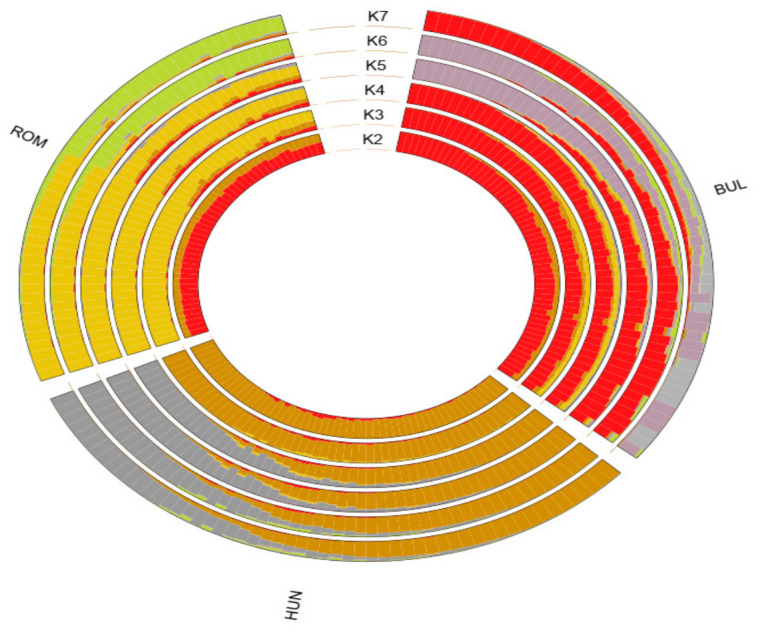
Admixture circle plot from K = 2 to 7 clusters for three Eastern European buffalo breeds. For breed abbreviations, see Figure 1.

The lowest CV error was observed at K = 7 (Supplementary Materials; Figure S1), indicating the best statistical fit. Since the dataset included only three buffalo breeds, the higher K values primarily reflect finer substructures within a single breed, rather than biologically distinct ancestral groups. The admixture analysis between the BUL, HUN, and ROM buffalo breeds from K 2 to 7 was visualized (Figure 2 and Supplementary Materials; Figure S2). At K = 2, the HUN buffalo breed was separated from the BUL and ROM buffalo breeds, whereas K = 3 separated the three breeds. Starting from K = 4, additional clusters primarily represented admixture and within-breed heterogeneity. Therefore, K = 2–3 was considered the most biologically informative representation of population structure. These findings are consistent with the PCA plot.

### 3.2. Signatures of Selection in BUL, HUN, and ROM Buffalo Breeds

**Figure 3:**
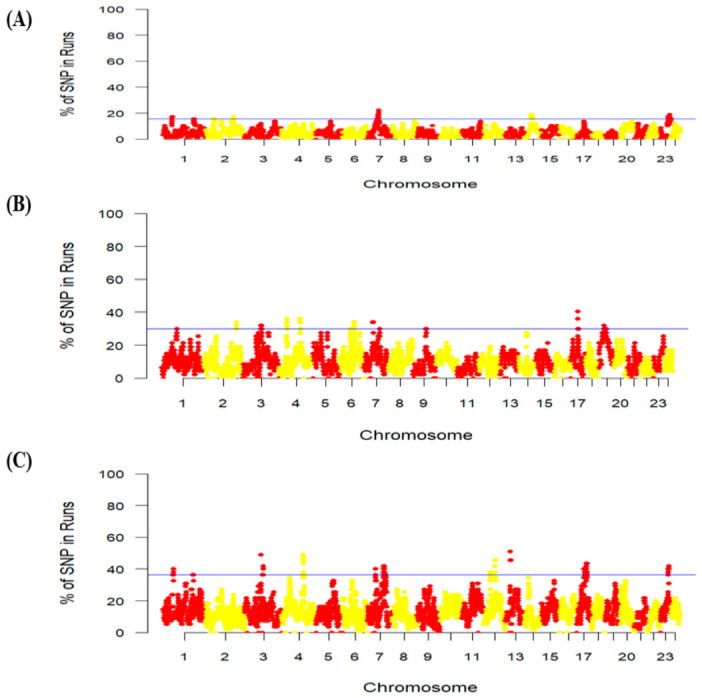
Manhattan plots of percentage of SNPs in run of homozygosity (ROH) signatures in Eastern European buffalo breeds. Horizontal blue lines mark the top 1% of SNPs with the highest ROH incidence in each breed: (**A**) ROH analysis for Bulgarian. (**B**) ROH analysis for Hungarian. (**C**) ROH analysis for Romanian.

Figure 3 shows genomic regions detected by the ROH method using the top 1% threshold in the BUL, HUN, and ROM buffalo breeds. In the BUL buffalo breed, we identified six ROH islands and 77 candidate genes subject to positive selection (Supplementary Materials; Table S1). Two genomic regions are located on chromosome 1 (41.21–44.75 Mb and 145.1–150.68 Mb). A genomic region on chromosome 3 (59.17–62.97), and two genomic regions on chromosome 7 (47.57–49.61 Mb and 51.24–56.29 Mb) were also identified. The highest number of genes (43) was detected on the ROH island (36.26–48.06 Mb) on chromosome 23. For the HUN buffalo breed, we detected 11 genomic regions, comprising 56 candidate genes as potential selection sweeps (Figure 3). We identified two ROH islands on chromosome 4 (97.05–97.47 Mb and 103.23–108.40 Mb), one on chromosome 6 (44.48–44.49 Mb), two on chromosome 7 (75.11–78.22 Mb and 84.29–88.38 Mb), one on chromosome 8 (57.46–59.38 Mb), one on chromosome 11 (74.90–75.49), one on chromosome 13 (43.05–45.04 Mb), one on chromosome 15 (50.58–51.23 Mb), one on chromosome 17 (45.73–50.87 Mb), and one on chromosome 20 (15.78–23.19 Mb) (Supplementary Materials; Table S1).

Five ROH islands, including 151 candidate genes, were revealed using the ROH approach in the ROM buffalo breed (Figure 3). These five ROH islands are located on chromosomes 2 (2.49–5.96 Mb), 3 (88.09–92.28 Mb), 6 (71.11–76.41 Mb), 17 (34.17–37.66 Mb), and 19 (14.81–40.44 Mb), involving 23, 14, 11, 4, and 99 candidate genes, respectively (Supplementary Materials; Table S1).

**Figure 4:**
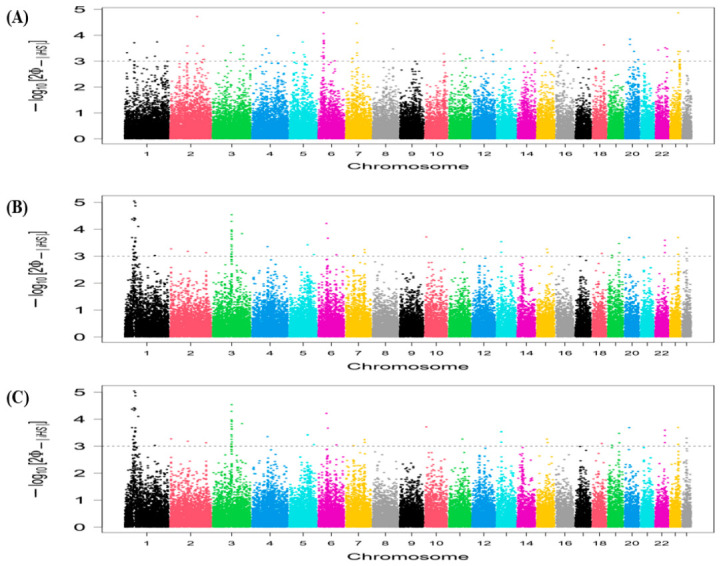
Manhattan plots of the genome-wide iHS test of selection signatures in Eastern European buffalo breeds. (**A**) iHS test for Bulgarian. (**B**) iHS test for Hungarian. (**C**) iHS test for Romanian. Horizontal dashed lines indicate the significance threshold applied to detect the outlier SNPs (−log10 (*p* value)  =  3).

The candidate genomic regions revealed based on the top 1% of iHS values within each breed are shown in Figure 4. A genomic region was detected as potential selective sweeps on chromosome 6 containing five candidate genes in the BUL buffalo breed (Supplementary Materials; Table S2). In the HUN buffalo breed, two genomic regions on chromosome 1 (41.67–43.65 Mb and 47.68–48.98 Mb), and one genomic region on chromosome 3 (86.40–88.59 Mb), comprising 7, 8, and 10 candidate genes, respectively, were identified. The iHS analysis revealed a single genomic region on chromosome 3 (86.40–88.59 Mb) containing 10 candidate genes that are subject to positive selection in the ROM buffalo breed (Supplementary Materials; Table S2).

**Figure 5:**
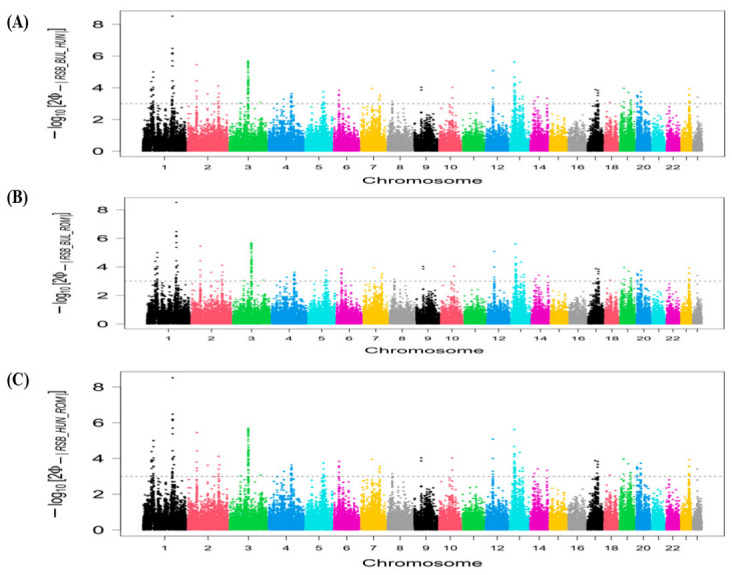
Manhattan plots of the genome-wide Rsb test of selection signatures in Eastern European buffalo breeds. (**A**) Rsb test for Bulgarian versus Hungarian. (**B**) Rsb test for Bulgarian versus Romanian. (**C**) Rsb test for Hungarian versus Romanian. Horizontal dashed lines indicate the significance threshold applied to detect the outlier SNPs (−log10 (*p* value)  =  3).

Applying the same threshold of the top 1% for the Rsb method to detect selection signatures between breed pairs, we identified 26 candidate genes within four genomic regions, as potential selective sweeps on four different chromosomes in the Bulgarian and Hungarian buffalo breeds (Figure 5). We found genomic regions on chromosomes 1 (136.04–137.82 Mb), 3 (86.44–88.49 Mb), 4 (104.97–107.41 Mb), and 13 (21.04–24.49 Mb) containing 2, 10, 8, and 6 candidate genes, respectively (Supplementary Materials; Table S3). When comparing the BUL buffalo breed vs. ROM buffalo breed, we revealed four genomic regions, harboring 26 candidate genes on four different chromosomes under positive selection (Figure 5). These genomic regions were discovered on chromosomes 1 (136.04–137.82 Mb), 3 (86.44–88.59 Mb), 4 (104.62–107.41 Mb), and 13 (21.04–24.49 Mb) comprising 2, 10, 8, and 6 candidate genes, respectively (Supplementary Materials; Table S3). In the comparison of the HUN buffalo breed vs. ROM buffalo breed, we detected 7 genomic regions and 28 candidate genes on different chromosomes that were subject to selection (Figure 5). These genomic regions are located on chromosomes 3 (88.59–89.65 Mb), 5 (92.61–93.57 Mb), 12 (30.74–31.50 Mb), 13 (39.69–40.37 Mb), 15 (74.26–74.92 Mb), and 17 (24.29–24.67 Mb and 38.70–39.89 Mb), which involve 14, 5, 2, 4, 1, 1, and 1 candidate genes, respectively (Supplementary Materials; Table S3).

**Figure 6:**
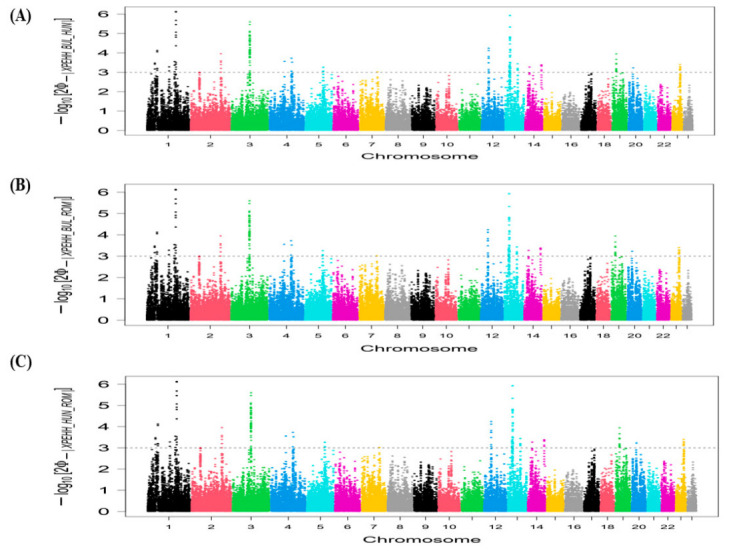
Manhattan plots of the genome-wide XP-EHH test of selection signatures in Eastern European buffalo breeds. (**A**) XP-EHH test for Bulgarian versus Hungarian. (**B**) XP-EHH test for Bulgarian versus Romanian. (**C**) XP-EHH test for Hungarian versus Romanian. Horizontal dashed lines indicate the significance threshold applied to detect the outlier SNPs (−log10 (*p* value)  =  3).

The XP-EHH test revealed three genomic regions including 17 candidate genes under positive selection on three different chromosomes for the comparison between the BUL buffalo breed and HUN buffalo breed (Figure 6). These genomic regions were identified on chromosomes 1 (137.65–137.82 Mb), 3 (86.44–88.28 Mb), and 13 (22.45–24.46 Mb), which contain 1, 10, and 6 candidate genes, respectively (Supplementary Materials; Table S4). In the BUL vs. ROM buffalo breeds, the XP-EHH test revealed four genomic regions, comprising 13 candidate genes on three different chromosomes (Figure 6). These genomic regions are located on chromosomes 1 (137.65–137.82 Mb), 3 (86.44–88.28 Mb), and 13 (23.07–24.46 Mb), and include 1, 10, and 2 candidate genes, respectively (Supplementary Materials; Table S4). Nine putative selection signals and 18 genes on different chromosomes were discovered in the HUN vs. ROM buffalo breeds (Figure 6). These genomic regions were detected on chromosomes 1 (94.53–95.23 Mb), 5 (92.61–94.19 Mb), 12 (30.70–31.33 Mb), 13 (39.73–40.37 Mb), 15 (74.26–74.92 Mb), 17 (24.29–24.67 Mb and 38.65–39.78 Mb), and 19 (67.02–69.08 Mb), containing 2, 5, 2, 4, 1, 1, 1, and 2 candidate genes, respectively, while the genomic region (29.49–29.90) on chromosome 15 did not contain any genes (Supplementary Materials; Table S4).

### 3.3. Overlapped Genomic Regions Detected Using at Least Two Methods Across Breeds

It is noteworthy that we found 10 overlapping genomic regions that were detected using at least two methods across lineages (Table 1). A genomic region ranging from 86.44 to 89.65 Mb on chromosome 3 was detected using the ROH, iHS, Rsb, and XP-EHH tests. In addition, an overlapping genomic region (135.17–137.82 Mb) on chromosome 1 was shared using both the Rsb and XP-EHH methods. We identified an overlapping genomic region (21.04–24.49 Mb) on chromosome 13 using the Rsb and XP-EHH approaches. These genomic regions and potential candidate genes are associated with economically important traits in buffalo.

The GO enrichment analysis revealed 6 biological processes, 12 cellular components, and 8 molecular function pathways (Supplementary Materials; Table S5). Although several GO terms, including positive regulation of the Wnt signaling pathway, synapse, and receptor complex, showed low nominal *p*-values, none remained significant after FDR correction. Therefore, these enrichments should be interpreted cautiously as exploratory signals rather than definitive functional associations.

## 4. Discussion

Understanding the genetic background and relationships among the BUL, HUN, and ROM buffalo breeds is critical to the management of breeding programs. Identifying the selection fingerprint is also crucial for breed conservation programs and for selecting buffalo breeds to improve production and environmental adaptation. Within these regions, we discovered several key candidate genes associated with growth, milk production, reproduction, and disease resistance. These candidate genes will be useful and can be used as marker-assisted selection in breeding programs in BUL, HUN, and ROM buffalo breeds.

### 4.1. Genetic Relationship and Admixture

The genetic background of buffalo breeds in Eastern European and their relationship with other European buffalo breeds and buffalo breeds worldwide have been studied [18,19]. To gain a clearer view of the genetic relationship between the BUL, HUN, and ROM buffalo breeds, we performed the PCA and admixture analyses. The PCA and admixture analyses showed a genetic variability among the BUL, HUN, and ROM buffalo breeds. These results are consistent with the findings of both [19,30]. This variation may be a result of the breeding management applied to each breed. The BUL breed was crossbred and then selected for milk production [1,4]. The ROM breed is well-adapted to the local environment [1], and this breed is subject to a selection program and has a high inbreeding coefficient based on ROHs [19]. It has been reported that the HUN breed was raised on small family farms for meat production, and no selection program was implemented for milk production [1]. Furthermore, ref. [19] found a high value for inbreeding and a large number of short ROHs in the HUN breed, indicating ancient inbreeding.

### 4.2. Selection Signatures in the BUL, HUN, and ROM Buffalo Breeds

Several genomic regions and potential candidate genes were identified using ROH, iHS, Rsb, and XP-EHH tests in the BUL, HUN, and ROM buffalo breeds. Interestingly, we highlighted ten overlapping genomic regions using at least two methods in the BUL, HUN, and ROM buffalo breeds (Table 1). A genomic region (41.67–43.65 Mb) on chromosome 1 was detected in the BUL buffalo breed using the ROH method and in the HUN buffalo breed using the iHS test. This genomic region was identified in the Italian Mediterranean buffalo using the ROH approach [31]. This region includes the *CSMD1* gene, which has been associated with fertility traits in German Holstein cattle [32] and with carcass traits in Hanwoo Korean cattle [33]. The genes *MYOM2* and *CLN8* have been linked to the growth and development of buffalo [34], chickens [35], and pigs [36]. The *NLGN1* and *NAALADL2* candidate genes were detected within a genomic region (136.04–137.82 Mb) on chromosome 1 in BUL vs. HUN and BUL vs. ROM using the Rsb and XP-EHH methods. This genomic region was reported in Chinese buffalo populations by Deng et al. [37]. The *NAALADL2* gene has been linked to immunity traits in cattle [38,39]. Another genomic region (86.44–88.49 Mb) on chromosome 3 was detected in the HUN and ROM buffalo breeds using the iHS test and in the BUL vs. HUN and BUL vs. ROM using the Rsb and XP-EHH tests. This region contains the candidate genes *FOCAD*, *MLLT3*, *SLC24A2*, *ACER2*, *DENND4C*, *SAXO1*, *HAUS6*, *PLIN2*, *RPS6*, and *RRAGA* (Table 1). The *SLC24A2* and *PLIN2* genes have been associated with milk production traits in cattle [40,41,42]. The *DENND4C* gene is related to intramuscular fat in rabbits [43] and metabolism for feet health in cattle [44]. The *PLIN2* gene also plays an important role in regulating progesterone synthesis in the corpus luteum of cattle [45]. The *RRAGA* gene plays a pivotal role in the growth and development of cows [46]. The *MLLT3* gene is involved in mastitis disease in cattle [47]. Using the Rsb method in HUN vs. ROM buffalo breeds, we discovered a genomic region (88.89–89.65 Mb) on chromosome 3, containing the *ADAMTSL1* gene, which is related to heat resistance in buffalo [48]. The *CCDC171* gene is significantly strongly associated with feed intake and residual feed intake traits in cattle [49] and the *CNTLN* gene is related to immunity functions in buffalo [50]. Using the Rsb and XP-EHH methods in HUN vs. ROM, we identified a genomic region (92.61–94.19 Mb) on chromosome 5 containing the *USP35* gene associated with milk production traits in cattle [51] and the *GAB2* gene, which was related to immunity traits in cattle [52]. A genomic region on chromosome 12 (30.74–31.50 Mb) contains the *LHCGR* and *FSHR* genes, which are associated with fertility and milk characteristics in cattle [53]. The *FSHR* gene is also strongly associated with reproductive traits and milk production in Egyptian buffalo [54,55,56,57]. Using the Rsb and XP-EHH approaches in BUL vs. HUN and BUL vs. ROM, we discovered a genomic region (21.04–24.49) on chromosome 13 harboring *GPR180*, *DCT*, *TGDS*, *SOX21*, *PC6*, and *GPC5* candidate genes (Supplementary Materials; Table S4). The *GPC5* gene has been associated with gestation length and other reproduction traits [58,59] as well as growth traits [60] in cattle. A genomic region on chromosome 13 (39.69–40.7 Mb) includes the *LMO7* gene, which is associated with reproductive traits in cattle [61]. This genomic region was detected in the Chinese buffalo crossbreds using the iHS test [37]. A genomic region on chromosome 15 (74.26–74.92 Mb) contains the *ST3GAL1* gene, which is associated with milk composition in goats [62]. There is a genomic region on chromosome 17 (24.29–24.67 Mb) that contains the *TMEM132C* gene and is associated with milk production traits in cattle [63].

## 5. Conclusions

Identifying the fingerprints of selection in the genomes of BUL, HUN, and ROM buffalo breeds provides information about the key genetic changes that shaped these important local breeds, which are specifically bred for milk and meat production. The use of the ROH, iHS, Rsb, and XP-EHH methods has revealed several candidate genes associated with milk production, reproduction, growth, and disease resistance characteristics in BUL, HUN, and ROM buffalo breeds. These identified candidate genes may provide preliminary candidate loci for future validation and for the design of conservation or breeding strategies. This work can serve as a basis for future assessment and tracking breeding programs, providing valuable support to farmers’ and breeders’ associations in Bulgaria, Hungary, and Romania.

## Figures and Tables

**Table 1:** Overlapping genomic regions detected using at least two methods in the BUL, HUN, and ROM buffalo breeds.

Test (Breed) ^1^	Chr ^2^	Position Mb	NSNPs ^3^	Length Mb	Genes	Trait
ROH (BUL); iHS (HUN)	1	41.21–44.75	81	3.54	***CSMD1***; *DLGAP2*; ***MYOM2***; ***CLN8***; *ERICH1*; *ARHGEF10*; *KBTBD11*	Reproduction; carcass traits; growth development
Rsb (BUL vs. HUN and BUL vs. ROM); XP-EHH (BUL vs. HUN and BUL vs. ROM)	1	136.04–137.82	16	1.77	*NLGN1*; ***NAALADL2***	Immune response
ROH (HUN); iHS (HUN and ROM); Rsb (BUL vs. HUN, BUL vs. ROM and HUN vs. ROM); XP-EHH (BUL vs. HUN and BUL vs. ROM)	3	86.40–88.59	17	2.19	*FOCAD*; ***MLLT3***; ***SLC24A2***; *ACER2*; ***DENND4C***; *SAXO1*; *HAUS6*; ***PLIN2***; *RPS6*; ***RRAGA***; *TRNAW-CCA*; *TRNAC-ACA*; *SNAPC3*; *FREM1*; ***ADAMTSL1***; *SH3GL2*; *CNTLN*; *BNC2*; *PSIP1*; *CCDC171*; *TTC39B*	Immune response; milk production; growth performance; reproduction; adaptation
Rsb (HUN vs. ROM); XP-EHH (HUN vs. ROM)	5	92.61–93.57	12	0.95	*TENM4*; *NARS2*; ***GAB2***; ***USP35***; *KCTD21*	Immune response; milk production
Rsb (HUN vs. ROM); XP-EHH (HUN vs. ROM)	12	30.70–31.33	19	0.76	***LHCGR***; ***FSHR***	Fertility
Rsb (BUL vs. HUN and BUL vs. ROM); XP-EHH (BUL vs. HUN and BUL vs. ROM)	13	21.04–24.49	24	3.38	*GPR180*; *DCT*; *TGDS*; *SOX21*; *PC6*; ***GPC5***	Reproduction
Rsb (HUN vs. ROM); XP-EHH (HUN vs. ROM)	13	39.69–40.37	17	0.68	***LMO7***; *COMMD6*; *UCHL3*; *TBC1D4*	Reproduction
Rsb (HUN vs. ROM); XP-EHH (HUN vs. ROM)	15	74.26–74.92	17	0.65	***ST3GAL1***	Milk composition
Rsb (HUN vs. ROM); XP-EHH (HUN vs. ROM)	17	24.29–24.67	13	0.38	***TMEM132C***	Milk production
Rsb (HUN vs. ROM); XP-EHH (HUN vs. ROM)	17	38.70–39.89	14	1.19	*ANKRD50*	--

^1^ BUL, Bulgarian; HUN, Hungarian; ROM, Romanian buffalo breeds. ROH, run of homozygosity; iHS, integrated haplotype score; Rsb; standardized log-ratio of the integrated site-specific extended haplotype homozygosity; XP-EHH, cross-population extended haplotype homozygosity. ^2^ Chr; chromosome. ^3^ NSNPs, number of single nucleotide polymorphisms. The genes highlighted in bold have been reported as genes associated with these economic traits.

## Data Availability

The genotyping data was obtained from the Dryad repository (https://doi.org/10.5061/dryad.9cnp5hqgc, accessed on 8 April 2021).

## Supplementary Materials

The following supporting information can be downloaded at: https://www.mdpi.com/article/10.3390/ani16101529/s1. Figure S1: Cross validation error; Figure S2: Bar plot Admixture analysis between the BUL, HUN and ROM buffalo breeds; Table S1: List of selected genomic regions detected by ROH method and candidate genes in each Buffalo breed; Table S2: List of selected genomic regions detected by iHS method and candidate genes in each Buffalo breed; Table S3: List of significant genomic regions detected by Rsb method and candidate genes between pairs of Buffalo breeds; Table S4: List of significant genomic regions detected by XP-EHH method and candidate genes between pairs of Buffalo breeds; Table S5: GO enrichment analysis of candidate genes in selection signatures.

## References

[1] Borghese A. (2005). Buffalo Production and Research.

[2] Low W.Y., Tearle R., Bickhart D.M., Rosen B.D., Kingan S.B., Swale T., Thibaud-Nissen F., Murphy T.D., Young R., Lefevre L. (2019). Chromosome-Level Assembly of the Water Buffalo Genome Surpasses Human and Goat Genomes in Sequence Contiguity. Nat. Commun..

[3] Mintoo A.A., Zhang H., Chen C., Moniruzzaman M., Deng T., Anam M., Emdadul Huque Q.M., Guang X., Wang P., Zhong Z. (2019). Draft Genome of the River Water Buffalo. Ecol. Evol..

[4] Borghese A., Moioli B. (2016). Buffalo: Mediterranean Region. Ref. Modul. Food Sci..

[5] Stepancheva T., Marinov I., Gergovska Z. (2024). Milking Temperament and Its Association with Test-Day Milk Yield in Bulgarian Murrah Buffaloes. Animals.

[6] Dimitrova I., Stancheva N., Genova K., Ilieva Y., Penchev P., Nenova R., Bozhilova-Sakova M. (2025). Water Buffalo: Origins and Genetic Diversity Associated with Economically Important Traits—A Review. Tradit. Mod. Vet. Med..

[7] Neață D.I., Vintilă T. (2023). The Importance for Conservation of the Romanian Buffalo Breed. Sci. Pap. Anim. Sci. Biotechnol..

[8] Popa D.C., Dudu A., Georgescu S.E., Burcea A., Popa R.A., Vidu L. (2020). Analysis of genetic diversity of Romanian buffalo–a preliminary study. Rom. Biotechnol. Lett..

[9] Minervino A.H.H., Zava M., Vecchio D., Borghese A. (2020). Bubalus Bubalis: A Short Story. Front. Vet. Sci..

[10] Jurco E.C., Onaciu G., Cuibus A., Cuibus L., Pop D. (2022). Contemporary Water Buffalo Farm Size in Romania and Actualized Milk Yields. Bull. Univ. Agric. Sci. Vet. Med. Cluj-Napoca Anim. Sci. Biotechnol..

[11] Karpati L. (1997). Buffaloes in Hungary. Bufalo Newlett.

[12] Zhang W., Yang M., Zhou M., Wang Y., Wu X., Zhang X., Ding Y., Zhao G., Yin Z., Wang C. (2020). Identification of Signatures of Selection by Whole-Genome Resequencing of a Chinese Native Pig. Front. Genet..

[13] Fan H., Wu Y., Qi X., Zhang J., Li J., Gao X., Zhang L., Li J., Gao H. (2014). Genome-Wide Detection of Selective Signatures in Simmental Cattle. J. Appl. Genet..

[14] Saravanan K.A., Rajawat D., Kumar H., Nayak S.S., Bhushan B., Dutt T., Panigrahi M. (2022). Signatures of Selection in Riverine Buffalo Populations Revealed by Genome-Wide SNP Data. Anim. Biotechnol..

[15] Biscarini F., Paolo C., Gaspa G., Gabriele M. (2019). Detect Runs of Homozygosity and Runs of Heterozygosity in Diploid Genomes.

[16] Sabeti P.C., Reich D.E., Higgins J.M., Levine H.Z.P., Richter D.J., Schaffner S.F., Gabriel S.B., Platko J.V., Patterson N.J., McDonald G.J. (2002). Detecting Recent Positive Selection in the Human Genome from Haplotype Structure. Nature.

[17] Sabeti P.C., Varilly P., Fry B., Lohmueller J., Hostetter E., Cotsapas C., Xie X., Byrne E.H., McCarroll S.A., Gaudet R. (2007). Genome-Wide Detection and Characterization of Positive Selection in Human Populations. Nature.

[18] Colli L., Milanesi M., Vajana E., Iamartino D., Bomba L., Puglisi F., Del Corvo M., Nicolazzi E.L., Ahmed S.S.E., Herrera J.R. (2018). V New Insights on Water Buffalo Genomic Diversity and Post-Domestication Migration Routes from Medium Density SNP Chip Data. Front. Genet..

[19] Noce A., Qanbari S., González-Prendes R., Brenmoehl J., Luigi-Sierra M.G., Theerkorn M., Fiege M.-A., Pilz H., Bota A., Vidu L. (2021). Genetic Diversity of Bubalus Bubalis in Germany and Global Relations of Its Genetic Background. Front. Genet..

[20] Nicolazzi E.L., Iamartino D., Williams J.L. (2014). AffyPipe: An Open-Source Pipeline for Affymetrix Axiom Genotyping Workflow. Bioinformatics.

[21] Chang C.C., Chow C.C., Tellier L.C.A.M., Vattikuti S., Purcell S.M., Lee J.J. (2015). Second-Generation PLINK: Rising to the Challenge of Larger and Richer Datasets. Gigascience.

[22] Alexander D.H., Novembre J., Lange K. (2009). Fast Model-Based Estimation of Ancestry in Unrelated Individuals. Genome Res..

[23] Milanesi M., Capomaccio S., Vajana E., Bomba L., Garcia J.F., Ajmone-Marsan P., Colli L. (2017). BITE: An R Package for Biodiversity Analyses. bioRxiv.

[24] Gorssen W., Meyermans R., Janssens S., Buys N. (2021). A Publicly Available Repository of ROH Islands Reveals Signatures of Selection in Different Livestock and Pet Species. Genet. Sel. Evol..

[25] Turner S.D. (2018). Qqman: An R Package for Visualizing GWAS Results Using QQ and Manhattan Plots. J. Open Source Softw..

[26] Gautier M., Klassmann A., Vitalis R. (2017). Rehh 2.0: A Reimplementation of the R Package Rehh to Detect Positive Selection from Haplotype Structure. Mol. Ecol. Resour..

[27] Browning S.R., Browning B.L. (2007). Rapid and Accurate Haplotype Phasing and Missing-Data Inference for Whole-Genome Association Studies by Use of Localized Haplotype Clustering. Am. J. Hum. Genet..

[28] Voight B.F., Kudaravalli S., Wen X., Pritchard J.K. (2006). A Map of Recent Positive Selection in the Human Genome. PLoS Biol..

[29] Quinlan A.R., Hall I.M. (2010). BEDTools: A Flexible Suite of Utilities for Comparing Genomic Features. Bioinformatics.

[30] Pauciullo A., Gaspa G., Versace C., Cosenza G., Piscopo N., Gu M., Coletta A., Hussain T., Seidavi A., Nicolae I. (2025). New Insights into Genetic Diversity and Differentiation of 11 Buffalo Populations Using Validated SNPs for Dairy Improvement. Genes.

[31] Liu S., Ma X., Hassan F., Gao T., Deng T. (2022). Genome-Wide Analysis of Runs of Homozygosity in Italian Mediterranean Buffalo. J. Dairy Sci..

[32] Sakhaeifar S., Yin T., König S. (2026). Longitudinal Genome-Wide Association Study for Female Fertility Traits in German Holstein Cattle. Anim. Genet..

[33] Lee S.H., Van Der Werf J.H.J., Kim N.K., Lee S.H., Gondro C., Park E.W., Oh S.J., Gibson J.P., Thompson J.M. (2011). QTL and Gene Expression Analyses Identify Genes Affecting Carcass Weight and Marbling on BTA14 in Hanwoo (Korean Cattle). Mamm. Genome.

[34] Mokhber M., Moradi-Shahrbabak M., Sadeghi M., Moradi-Shahrbabak H., Stella A., Nicolzzi E., Rahmaninia J., Williams J.L. (2018). A Genome-Wide Scan for Signatures of Selection in Azeri and Khuzestani Buffalo Breeds. BMC Genom..

[35] Jin Y., Tan X., Liu L., Li J., Dong J., Huang M., Zhao A., Wang D. (2026). Comparative Analysis of the Transcriptome of the Chicken Breast Muscle at Different Developmental Stages. Animals.

[36] Xiong H., Zhang Y., Zhao Z. (2024). Investigation of Single Nucleotide Polymorphisms in Differentially Expressed Genes and Proteins Reveals the Genetic Basis of Skeletal Muscle Growth Differences between Tibetan and Large White Pigs. Anim. Biosci..

[37] Deng T.X., Ma X.Y., Lu X.R., Duan A.Q., Shokrollahi B., Shang J.H. (2022). Signatures of Selection Reveal Candidate Genes Involved in Production Traits in Chinese Crossbred Buffaloes. J. Dairy Sci..

[38] Zhang S., Yao Z., Li X., Zhang Z., Liu X., Yang P., Chen N., Xia X., Lyu S., Shi Q. (2022). Assessing Genomic Diversity and Signatures of Selection in Pinan Cattle Using Whole-Genome Sequencing Data. BMC Genom..

[39] Zhong Z., Wang Z., Xie X., Pan D., Su Z., Fan J., Xiao Q., Sun R. (2024). Insights into Adaption and Growth Evolution: Genome–Wide Copy Number Variation Analysis in Chinese Hainan Yellow Cattle Using Whole–Genome Re–Sequencing Data. Int. J. Mol. Sci..

[40] Da Cruz A.S., Silva D.C., Minasi L.B., de Farias Teixeira L.K., Rodrigues F.M., da Silva C.C., do Carmo A.S., da Silva M.V.G.B., Utsunomiya Y.T., Garcia J.F. (2021). Single-Nucleotide Polymorphism Variations Associated with Specific Genes Putatively Identified Enhanced Genetic Predisposition for 305-Day Milk Yield in the Girolando Crossbreed. Front. Genet..

[41] Persichilli C., Senczuk G., Mastrangelo S., Marusi M., van Kaam J.-T., Finocchiaro R., Di Civita M., Cassandro M., Pilla F. (2023). Exploring Genome-Wide Differentiation and Signatures of Selection in Italian and North American Holstein Populations. J. Dairy Sci..

[42] Li Y.H., Zhou H., Cheng L., Zhao J., Hickford J.G.H. (2020). Variation in *PLIN2* and Its Association with Milk Traits and Milk Fat Composition in Dairy Cows. J. Agric. Sci..

[43] Varona L., Blasco A. (2020). The Effect of Divergent Selection for Intramuscular Fat on the Domestic Rabbit Genome. Animal.

[44] Zsolnai A., Bognár L., Bene S.A., Kőrösi Z.J., Rózsa L., Szabó F., Anton I. (2026). Effects of Single-Nucleotide Polymorphisms on the Estimated Breeding Values for Feet in Holstein-Friesian Cows in Hungary. Animals.

[45] Plewes M.R., Talbott H.A., Schott M.B., Wood J.R., Cupp A.S., Davis J.S. (2024). Unraveling the Role of Lipid Droplets and Perilipin 2 in Bovine Luteal Cells. FASEB J..

[46] Cunningham-Hollinger H.C., Kuehn L.A., Cammack K.M., Hales K.E., Oliver W.T., Crouse M.S., Chen C., Freetly H.C., Lindholm-Perry A.K. (2021). Transcriptome Profiles of the Skeletal Muscle of Mature Cows during Feed Restriction and Realimentation. BMC Res. Notes.

[47] Ma S., Tong C., Ibeagha-Awemu E.M., Zhao X. (2019). Identification and Characterization of Differentially Expressed Exosomal MicroRNAs in Bovine Milk Infected with Staphylococcus Aureus. BMC Genom..

[48] Bian C., Luo Y., Li J., Cheng H., He F., Duan H., Ahmed Z., Lei C., Yi K. (2024). Inference of Genetic Diversity, Population Structure, and Selection Signatures in Xiangxi White Buffalo of China Through Whole-Genome Resequencing. Genes.

[49] Santana M.H., Utsunomiya Y.T., Neves H.H., Gomes R.C., Garcia J.F., Fukumasu H., Silva S.L., Oliveira Junior G.A., Alexandre P.A., Leme P.R. (2014). Genome-Wide Association Analysis of Feed Intake and Residual Feed Intake in Nellore Cattle. BMC Genet..

[50] Liu S., Kang X., Catacchio C.R., Liu M., Fang L., Schroeder S.G., Li W., Rosen B.D., Iamartino D., Iannuzzi L. (2019). Computational Detection and Experimental Validation of Segmental Duplications and Associated Copy Number Variations in Water Buffalo (*Bubalus bubalis*). Funct. Integr. Genom..

[51] Zan Y., Li J., Song F., Tang Y., Xin Y., Zhang Z., Wang L., Zhang L., Du L., Yuan Z. (2025). Genome-Wide Identification and Characterization of the Ubiquitin-Specific Protease (USP) Gene Family in Cattle: Primary Analysis of Muscle-Specific USP Genes and Their Influence on Myogenesis. BMC Genom..

[52] Matenchi Y.P., Hegarty M. (2025). Genomic and Regulatory Basis of Adaptation in Cameroonian Gudali and Simgud Cattle. bioRxiv.

[53] Abeygunawardana D.I., Ranasinghe R.M.S.B.K., De Silva S.N.T., Deshapriya R.M.C., Gamika P.A., Rajapakse J. (2023). Effect of LHCGR and FSHR Gene Polymorphisms on Fertility Traits and Milk Yield of Cross-Bred Dairy Cows in Sri Lanka. Anim. Biotechnol..

[54] Ramadan S., Saker A.-A., Shafik B. (2020). DNA Polymorphism of FSHR Gene and Its Association with Infertility Traits in Egyptian Buffaloes. Benha Vet. Med. J..

[55] Sallam E., Mohammed L., Darwish S., Hegazy M., El-Naby A., Azzam A. (2022). Detection of FSHR Gene Variants Associated with Sperm Motility in Egyptian Buffalo Bulls. Benha Vet. Med. J..

[56] Zaghloul A.R., Khalil M.H., Iraqi M.M., Amin A.M.S., Abousoliman I., EL Nagar A.G. (2025). Genetic Analyses and Molecular Associations of FSHR and GH Genes for Semen Traits in Egyptian Buffalo. BMC Vet. Res..

[57] Zaghloul A.R., Khalil M.H., Iraqi M.M., Amin A.M.S., EL Nagar A.G. (2025). Genetic Polymorphisms of PRL, DGAT1, FSHR, and GH Genes and Their Associations with Milk and Reproduction Traits in Egyptian Buffalo (*Bubalus bubalis*). BMC Genom..

[58] Purfield D.C., Evans R.D., Carthy T.R., Berry D.P. (2019). Genomic Regions Associated with Gestation Length Detected Using Whole-Genome Sequence Data Differ Between Dairy and Beef Cattle. Front. Genet..

[59] Dubon M.A.C., Pedrosa V.B., Feitosa F.L.B., Costa R.B., de Camargo G.M.F., Silva M.R., Pinto L.F.B. (2021). Identification of Novel Candidate Genes for Age at First Calving in Nellore Cows Using a SNP Chip Specifically Developed for Bos Taurus Indicus Cattle. Theriogenology.

[60] Purfield D.C., Evans R.D., Berry D.P. (2020). Breed- and Trait-Specific Associations Define the Genetic Architecture of Calving Performance Traits in Cattle. J. Anim. Sci..

[61] Raja T.V., Alex R., Singh U., Kumar S., Das A.K., Sengar G., Singh A.K., Ghosh A., Saha S., Mitra A. (2023). Genome-Wide Identification and Annotation of SNPs for Economically Important Traits in Frieswal^TM^, Newly Evolved Crossbred Cattle of India. 3 Biotech.

[62] Crisà A., Claps S., Moioli B., Marchitelli C. (2019). Identification of the Complete Coding CDNAs and Expression Analysis of B4GALT1, LALBA, ST3GAL5, ST6GAL1 in the Colostrum and Milk of the Garganica and Maltese Goat Breeds to Reveal Possible Implications for Oligosaccharide Biosynthesis. BMC Vet. Res..

[63] Sukhija N., Malik A.A., Devadasan J.M., Dash A., Bidyalaxmi K., Ravi Kumar D., Kousalaya Devi M., Choudhary A., Kanaka K.K., Sharma R. (2024). Genome-Wide Selection Signatures Address Trait Specific Candidate Genes in Cattle Indigenous to Arid Regions of India. Anim. Biotechnol..

