# Supplementary material for: Genomic Survey of Selection Footprints in Three Buffalo Breeds from Eastern Europe
Source: Animals (Basel). 2026 May 16;16(10):1529. doi: 10.3390/ani16101529 (PMC13203351; doi:10.3390/ani16101529)
Supplement: Supplementary file 1 [file animals-16-01529-s001.zip › animals-4323090-supplementary.pdf]

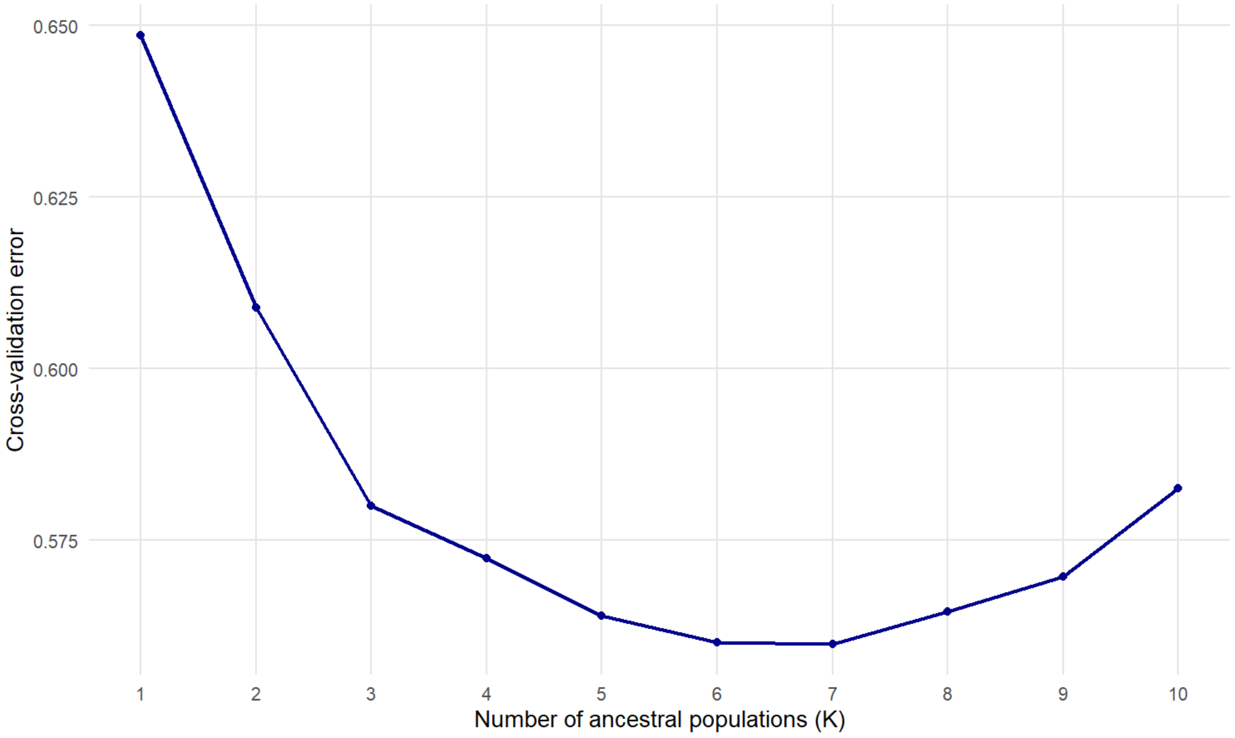

Figure S1. Cross validation error

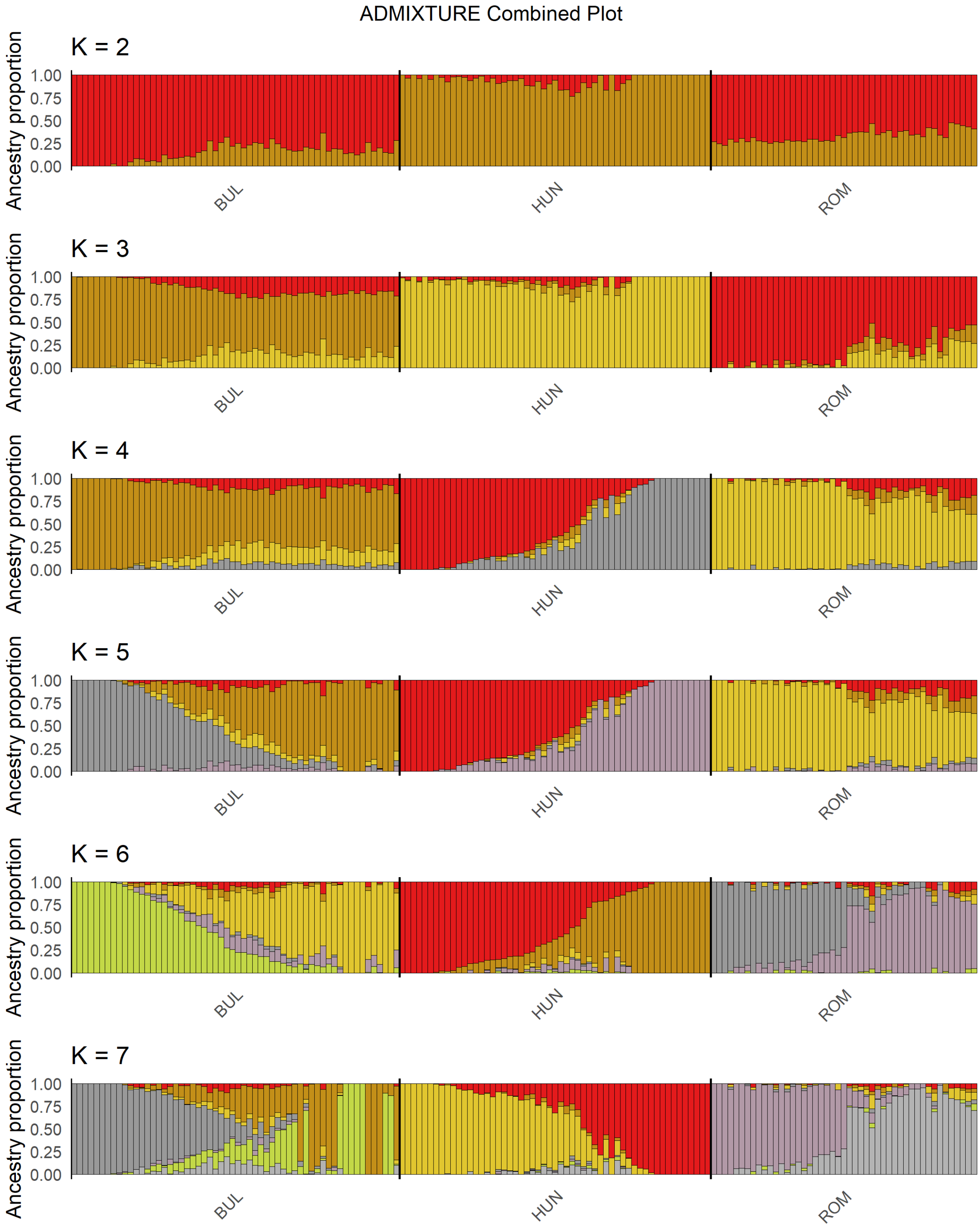

Figure S2. Bar plot Admixture analysis between the BUL, HUN and ROM buffalo breeds.

Table S1: List of selected genomic regions detected by ROH method and candidate genes in each Buffalo breed

Breed	Chr	Start	End	Lenght (bp)	n°SNPs	Genes
**BUL**	1	41211311	44752486	3541175	81	*CSMD1; DLGAP2; MYOM2; CLN8; ERICH1; ARHGEF10; KBTBD11*
	1	145105204	150682548	5577344	160	*SI; SPTSSB; BCHE; OTOL1; SLITRK3*
	3	59175966	62970284	3794318	88	*CA10*
	7	47578553	49619984	2041431	50	*CHIC2; GSX2; SCFD2; FIP1L1; LNX1; RASL11B; USP46; SPATA18; SGCB; LRRC66*
	7	51241751	56294318	5052567	144	*GABRB1; ATP10D; MMD8; GABRA2; GABRG1; KCTD8; GUF1; GNPDA2; YIPF7; ATP8A1; GRXCR1; SHISA3*
	23	36264407	48065055	11800648	328	*GRK5; PLPP4; ATE1; TACC2; CPXM2; LHPP; CTBP2; ADAM12; DOCK1; PTPRE; C23H10orf90; INSYN2; FANK1; CTBP2; ZRANB1; LHPP; FAM53B; CHST15; CPXM2; GPR26; MKI67; CLRN3; OAT; PDZD8; SLC18A2; RAB11FIP2; CACUL1; SFXN4; RGS10; TIAL1; BAG3; SEC23IP; PLPP4; WDR11; FGFR2; BTBD16; PLEKHA1; HTRA1; PSTK; IKZF5; ACADSB; BUB3*
**HUN**	4	97056874	97473345	416471	13	*NUDT4*
	4	103231265	108404811	5173546	133	*MGAT4C; NTS; RASSF9; LRRIQ1; TSPAN19; SLC6A15; TMTC2; METTL25*
	6	44480518	44935740	455222	13	--
	7	75111457	78226918	3115461	88	*ADGRA3; KCNIP4; SLIT2; PACRGL*
	7	84295772	88385293	4089521	95	*UGT8; TRNAK-UUU; ARSJ; CAMK2D; ANK2*
	8	57467975	59388149	1920174	56	*IMMP2L; THAP5*
	11	74909102	75495490	586388	15	*EMC7; EMC4; KATNBL1; SLC12A6; NUTM1; NOP10; LPCAT4*
	13	43050043	45045423	1995380	55	*PIBF1; BORA; DIS3; MZT1; DACH1*
	15	50588224	51232466	644242	21	*CPA6; ARFGEF1; CSPP1; COPS5; PPP1R42; TCF24*
	17	45734615	50871152	5136537	130	*PCDH10; PABPC4L*
	20	15785661	23198470	7412809	173	*LRFN5; FBXO33; PNN; GEMIN2; TRAPPC6B; SEC23A; CLEC14A; SSTR1; FOXA1; PAX9; NKX2-8; MBIP; MIA2; TTC6; MIPOL1; SLC25A21*

Cont. Table S1: List of selected genomic regions detected by ROH method and candidate genes in each Buffalo breed

Breed	Chr	Start	End	Lenght (bp)	n°SNPs	Genes
**ROM**	2	2491165	5961787	3470622	62	*TRNAF-GAA; CDYL; TRNAC-ACA; PPP1R3G; RPP40; LYRM4; FARS2; NRN1; F13A1; LY86; RREB1; SSR1; RIOK1; CAGE1; TRNAP-AGG; TRNAE-UUC; SNRNP48; DSP; BMP6; TXNDC5; BLOC1S5; EEF1E1; SLC35B3*
	3	88096927	92289316	4192389	116	*HAUS6; RRAGA; TRNAW-CCA; TRNAC-ACA; SNAPC3; FREM1; SAXO1; ADAMTSL1; SH3GL2; CNTLN; BNC2; PSIP1; CCDC171; TTC39B*
	6	71115556	76410417	5294861	152	*NEGR1; ZRANB2; PTGER3; CTH; ANKRD13C; SRSF11; LRRC40; LRRC7; DEPDC1; RPE65; WLS*
	17	34178784	37662096	3483312	88	*TRNAC-ACA; FSTL5; TRPC3; KIAA1109*
	19	14816805	40440937	25624132	692	*HTR1A; DIMT1; KIF2A; TRNAW-CCA; SMIM15; ERCC8; ELOVL7; DEPDC1B; RNF180; LOC123330542; IPO11; LOC112580614; ZSWIM6; NDUFAF2; PDE4D; TRNAC-GCA; RAB3C; GAPT; PLK2; TRNAC-ACA; MIER3; GPBP1; ACTBL2; SETD9; MAP3K1; ANKRD55; PLPP1; SNX18; GZMA; MTREX; SLC38A9; IL31RA; GPX8; IL6ST; CDC20B; GZMK; ESM1; DHX29; MCIDAS; CCNO; ARL15; ITGA1; ITGA2; FST; PELO; NDUFS4; MOCS2; ISL1; PARP8; EMB; HCN1; FGF10; MRPS30; NNT; C19H5orf34; ZNF13; NIM1K; HMGCS1; CCDC152; CCL28; SELENOP; GHR; PLCXD3; C19H5orf51; C6; C7; CARD6; OXCT1; PRKAA1; TTC33; FBXO4; PTGER4; MROH2B; RPL37; WDR70; CPLANE1; NUP155; NIPBL; RANBP3L; LMBRD2; CAPSL; NADK2 ; SLC1A3; IL7R; SKP2; SPEF2; PRLR; DNAJC21; ADAMTS12; TARS; RAI14; TTC23L; AGXT2; RAD1; SLC45A2; AMACR; C1QTNF3; RXFP3; BRIX1*

Chr = chromosome; BUL = Bulgarian; HUN = Hungarian; ROM = Romanian; ROH = runs of homozygosity.

Table S2: List of significant genomic regions detected by iHS method and candidate genes in each buffalo breed

Breed	Chr	Start	End	Length (bp)	n°SNPs	iHS score	LOGPVALUE	Genes
**BUL**	6	23672355	25150876	1478521	11	-3.8975	4.0124	*WARS2; TBX15; SPAG17; GDAP2; WDR3*
**HUN**	1	41677163	43654680	1977517	14	4.4444	5.0548	*CSMD1; DLGAP2; MYOM2; CLN8; ERICH1; ARHGEF10; KBTBD11*
	1	47684946	48980402	1295456	12	4.4353	5.0364	*EVA1C; URB1; MRAP; MIS18A; HUNK; SCAF4; SOD1; TIAM1*
	3	86402435	88595971	2193536	17	4.1637	4.5042	*FOCAD; MLLT3; SLC24A2; ACER2; DENND4C; SAXO1; HAUS6; PLIN2; RPS6; RRAGA*
**ROM**	3	86402435	88595971	2193536	16	3.6949	3.6576	*FOCAD; MLLT3; SLC24A2; ACER2; DENND4C; SAXO1; HAUS6; PLIN2; RPS6; RRAGA*

Chr = chromosome; BUL = Bulgarian; HUN = Hungarian; ROM = Romanian; iHS = integrated haplotype score.

Table S3: List of significant genomic regions detected by Rsb method and candidate genes between pairs of Buffalo breeds

Group	Chr	Start	End	Length (bp)	n°SNPs	Rsb score	LOGPVALUE	DOS	Genes
**BUL vs HUN**	1	136049918	137829033	1779115	16	-5.8820	8.3922	HUN	*NLGN1; NAALADL2*
	3	86440124	88493413	2053289	46	-4.6854	5.5537	HUN	*FOCAD; MLLT3; SLC24A2; ACER2; RPS6; DENND4C; SAXO1; HAUS6; PLIN2; RRAGA*
	4	104979951	107418507	2438556	11	-3.5727	3.4519	HUN	*MGAT4C; NTS; RASSF9; LRRIQ1; TSPAN19; SLC6A15; TMTC2; METTL25*
	13	21043273	24491258	3447985	26	-4.6741	5.5298	ROM	*GPR180; DCT; TGDS; SOX21; GPC5; GPC6*
**BUL vs ROM**	1	135174530	137829033	2654503	18	-5.9330	8.5265	ROM	*NLGN1; NAALADL2*
	3	86440124	88595971	2155847	44	-4.5025	5.1730	ROM	*FOCAD; MLLT3; SLC24A2; ACER2; DENND4C; SAXO1; HAUS6; PLIN2; RPS6; RRAGA*
	4	104627695	107418507	2790812	13	-3.7498	3.7521	ROM	*MGAT4C; NTS; RASSF9; LRRIQ1; TSPAN19; SLC6A15; TMTC2; METTL25*
	13	21102877	24491258	3388381	24	-4.5118	5.1919	ROM	*GPR180; DCT; TGDS; SOX21; PC6; GPC5*
**HUN vs ROM**	3	88595971	89659640	1063669	10	-4.9733	6.1817	ROM	*HAUS6; RRAGA; TRNAW-CCA; TRNAC-ACA; SNAPC3; FREM1; SAXO1; ADAMTSL1; SH3GL2; CNTLN; BNC2; PSIP1; CCDC171; TTC39B*
	5	92619216	93578356	959140	12	4.1859	4.5467	HUN	*TENM4; NARS2; GAB2; USP35; KCTD21*
	12	30744781	31509637	764856	19	-5.8135	8.2133	ROM	*LHCGR; FSHR*
	13	39696216	40378126	681910	17	5.3378	7.0265	HUN	*LMO7; COMMD6; UCHL3; TBC1D4*
	15	74265814	74925804	659990	17	-4.7035	5.5922	ROM	*ST3GAL1*
	17	24298075	24674935	376860	13	-4.5943	5.3623	ROM	*TMEM132C*
	17	38706622	39892536	1185914	14	-5.5437	7.5285	ROM	*ANKRD50*

Chr = chromosome; BUL = Bulgarian; HUN = Hungarian; ROM = Romanian; Rsb = standardized log-ratio of the integrated site-specific extended haplotype homozygosity; DOF = Direction of selection.

Table S4: List of significant genomic regions detected by XP-EHH method and candidate genes between pairs of Buffalo breeds

Group	Chr	Start	End	Length (bp)	n°SNPs	XP-EHH score	LOGPVALUE	DOF	Genes
**BUL vs HUN**	1	137654304	137829033	174729	13	-4.9254	6.0747	HUN	*NAALADL2*
	3	86440124	88288478	1848354	47	-4.6823	5.5471	HUN	*FOCAD; MLLT3; SLC24A2; ACER2; DENND4C; SAXO1; HAUS6; PLIN2; RPS6; RRAGA*
	13	22459940	24464526	2004586	26	-4.8343	5.8742	HUN	*GPR180; DCT; TGDS; SOX21; GPC6; GPC5*
**BUL vs ROM**	1	137654304	137829033	174729	13	-4.9706	6.1756	ROM	*NAALADL2*
	3	86440124	88288478	1848354	46	-4.4241	5.0139	ROM	*FOCAD; MLLT3; SLC24A2; ACER2; DENND4C; SAXO1; HAUS6; PLIN2; RPS6; RRAGA*
	13	23077907	24464526	1386619	23	-4.8588	5.9277	ROM	*GPC6; GPC5*
**HUN vs ROM**	1	94539363	95230346	690983	10	-3.7470	3.7473	ROM	*ALCAM; CBLB*
	5	92619216	94192862	1573646	16	4.5201	5.2089	HUN	*TENM4; NARS2; GAB2; USP35; KCTD21*
	12	30703751	31338374	634623	16	-6.3192	9.5801	ROM	*LHCGR; FSHR*
	13	39735094	40378126	643032	15	5.5275	7.4883	HUN	*LMO7; TBC1D4; COMMD6; UCHL3*
	15	29496191	29907349	411158	13	-3.9801	4.1618	ROM	
	15	74265814	74925804	659990	14	-4.7263	5.6407	ROM	*ST3GAL1*
	17	24298075	24674935	376860	13	-4.9480	6.1251	ROM	*TMEM132C*
	17	38650275	39786247	1135972	14	-4.8982	6.0144	ROM	*ANKRD50*
	19	67025650	69080511	2054861	17	4.9364	6.0992	ROM	*ADAMTS16; ICE1*

Chr = chromosome; BUL = Bulgarian; HUN = Hungarian; ROM = Romanian; XP-EHH = cross-population extended haplotype homozygosity; DOF = Direction of selection.

Table S5. GO enrichment analysis of candidate genes in selection signatures

Category	Term	Count	Total Gene	Pop Hits	Pop Total	Gene %	PValue	Bonferroni	FDR	Fisher Exact	Genes
Biological Process	positive regulation of Wnt signaling pathway	5	226	24	15264	0.38	0.0003	0.427	0.557	0.000026	*ZRANB1; DEPDC1B; FAM53B; GPC5; FGF10*
Biological Process	regulation of signaling	21	226	817	15264	1.61	0.0182	1	1	0.00666	*ARFGEF1; SLC24A2; ZRANB1; NLGN1; FST; DENND4C; HTR1A; LY86; LMO7; KCTD8; MRAP; FSTL5; RRAGA; DEPDC1B; GRK5; RGS10; FAM53B; GPC5; IL7R; EEF1E1; GPC6; FGF10*
Biological Process	positive regulation of canonical Wnt signaling pathway	3	226	19	15264	0.23	0.0314	1	1	0.0028	*FAM53B; GPC5; FGF10*
Biological Process	regulation of cell communication	20	226	815	15264	1.54	0.0326	1	1	0.0127	*ARFGEF1; SLC24A2; ZRANB1; NLGN1; FST; DENND4C; HTR1A; LY86; KCTD8; MRAP; FSTL5; RRAGA; DEPDC1B; GRK5; RGS10; FAM53B; GPC5; IL7R; EEF1E1; GPC6; FGF10*
Biological Process	regulation of signal transduction	18	226	718	15264	1.38	0.0371	1	1	0.0259	*ARFGEF1; ZRANB1; FST; DENND4C; LY86; KCTD8; MRAP; FSTL5; RRAGA; DEPDC1B; GRK5; RGS10; FAM53B; GPC5; IL7R; EEF1E1; GPC6; FGF10*

Cont. Table S5. GO enrichment analysis of candidate genes in selection signatures

Category	Term	Count	Total Gene	Pop Hits	Pop Total	Gene %	PValue	Bonferroni	FDR	Fisher Exact	Genes
Biological Process	regulation of Wnt signaling pathway	5	226	88	15264	0.38	0.0411	1	1	0.0109	*ZRANB1; DEPDC1B; FAM53B; GPC5; FGF10*
Cellular Component	synapse	15	214	393	14743	1.15	0.0017	0.857	1	0.00309	*GABRA2; RAB3C; NLGN1; GABRB1; NTS; GABRG1; TIAM1; PLCXD3; ACTBL2; LRRC7; SLITRK3; GPC6; DLGAP2; SH3GL2; SLC18A2*
Cellular Component	receptor complex	9	214	198	14743	0.69	0.0081	0.996	1	0.00709	*GABRA2; GABRB1; ITGA2; ITGA1; IL31RA; IL6ST; PRLR; FGFR2; GABRG1*
Cellular Component	centrosome	8	214	162	14743	0.61	0.0092	0.996	1	0.00677	*ARHGEF10; C23H10ORF90; PLK2; CSPP1; PIBF1; MZT1; CCNO; CNTLN*
Cellular Component	cell surface	12	214	337	14743	0.92	0.0099	0.857	1	0.0133	*GHR; NLGN1; ITGA2; I TGA1; IL31RA; CLEC14A; GPC5; ADGRA3; IL7R; IL6ST; PRLR; GPC6*
Cellular Component	external side of plasma membrane	9	214	213	14743	0.69	0.0124	1	1	0.0112	*GHR; ITGA2; ITGA1; IL31RA; CLEC14A; ADGRA3; IL7R; IL6ST; PRLR*
Cellular Component	plasma membrane	44	214	2135	14743	3.38	0.0137	1	1	0.0305	*PTGER4; RAB3C; NLGN1; NRN1; ATP8A1; PCDH10; ATP10D; PTGER3; SLC1A3; CLEC14A; GHR; C6; C7; SGCB; SLITRK3; GPC5; EMB; RAB11FIP2; GPC6; GABRA2; DSP; BCHE; LHCGR; SLC6A15; ITGA2; ITGA1; KCNIP4; HTR1A; IL31RA; ADGRA3; ANK2; PRLR; GABRG1; MRAP; TIAM1; SNX18; LRRC7; FSHR; IL6ST; IL7R; DOCK1; FGFR2*

Cont. Table S5. GO enrichment analysis of candidate genes in selection signatures

Category	Term	Count	Total Gene	Pop Hits	Pop Total	Gene %	PValue	Bonferroni	FDR	Fisher Exact	Genes
Cellular Component	cell periphery	47	214	2331	14743	3.61	0.0152	1	1	0.0335	*PTGER4; RAB3C; NLGN1; NRN1; ATP8A1; PCDH10; ATP10D; PTGER3; SLC1A3; CLEC14A; ADAMTS12; ADAMTSL1; GHR; ADAMTS16; C6; C7; SGCB; SLITRK3; GPC5; EMB; RAB11FIP2; GPC6; GABRA2; DSP; BCHE; LHCGR; SLC6A15; ITGA2; ITGA1; KCNIP4; HTR1A; IL31RA; ADGRA3; ANK2; PRLR; GABRG1; MRAP; TIAM1; SNX18; LRRC7; FSHR; IL6ST; IL7R; DOCK1; FGFR2*
Cellular Component	microtubule organizing center	9	214	241	14743	0.69	0.024	1	1	0.0232	*ARHGEF10; C23H10ORF90; PLK2; CSPP1; SAXO1; PIBF1; MZT1; CCNO; CNTLN*
Cellular Component	GABA receptor complex	3	214	19	14743	0.23	0.0302	1	1	0.0039 5	*GABRA2; GABRB1; GABRG1*
Cellular Component	GABA-A receptor complex	3	214	19	14743	0.23	0.0302	1	1	0.0039 5	*GABRA2; GABRB1; GABRG1*
Cellular Component	neuron projection	12	214	409	14743	0.92	0.0356	1	1	0.0492	*GABRA2; GRXCR1; GABRB1; CAMK2D; NEGR1; TENM4; ACTBL2; SSTR1; EMB; NTS; SLC18A2; GABRG1*
Cellular Component	cell junction	16	214	649	14743	1.23	0.0496	1	1	0.0317	*DSP; GABRA2; RAB3C; NLGN1; GABRB1; NTS; GABRG1; TIAM1; PLCXD3; ACTBL2; LRRC7; SLITRK3; GPC6; DLGAP2; SH3GL2; SLC18A2*

Cont. Table S5. GO enrichment analysis of candidate genes in selection signatures

Category	Term	Count	Total Gene	Pop Hits	Pop Total	Gene %	PValue	Bonferroni	FDR	Fisher Exact	Genes
Molecular Function	solute:monoatomic cation symporter activity	5	226	73	15847	0.38	0.0201	1	1	0.00276	*SLC45A2; SLC6A15; SLC1A3; SLC12A6; SLC18A2*
Molecular Function	GABA-A receptor activity	3	226	19	15847	0.23	0.0293	1	1	0.00188	*GABRA2; GABRB1; GABRG1*
Molecular Function	cytokine receptor activity	5	226	83	15847	0.38	0.0304	1	1	0.0048	*GHR; IL31RA; IL7R; IL6ST; PRLR*
Molecular Function	GABA receptor activity	3	226	22	15847	0.23	0.0385	1	1	0.00289	*GABRA2; GABRB1; GABRG1*
Molecular Function	catalytic activity, acting on RNA	9	226	275	15847	0.69	0.0429	1	1	0.0107	*ATE1; DIMT1; DIS3; NARS2; RPP40; MTREX; DHX29; WARS2; FARS2*
Molecular Function	catalytic activity, acting on a tRNA	5	226	95	15847	0.38	0.0463	1	1	0.00844	*ATE1; NARS2; RPP40; WARS2; FARS2*
Molecular Function	ubiquitin-like protein peptidase activity	5	226	96	15847	0.38	0.0478	1	1	0.00882	*USP35; USP46; ZRANB1; COPS5; UCHL3*
Molecular Function	immune receptor activity	5	226	104	15847	0.38	0.0608	1	1	0.0122	*GHR; IL31RA; IL7R; IL6ST; PRLR*
